# Comparative analysis of Buruli ulcer in Ghana and Côte d’Ivoire: A cross-sectional study

**DOI:** 10.1371/journal.pntd.0013912

**Published:** 2026-01-12

**Authors:** Elizabeth Gyamfi, Magdalene Dogbe, Mabel Sarpong-Duah, Edwin Sakyi Kyei-Baffour, Kwabena Owusu Boateng, Daisy Awuku Asante, Joshua Adeebute Ayelazuno, Charles Quaye, Abel Adjet Afouda, Lydia Mosi

**Affiliations:** 1 West African Centre for Cell Biology of Infectious Pathogens, University of Ghana, Legon, Accra, Ghana; 2 Department of Biochemistry, Cell and Molecular Biology, College of Basic and Applied Sciences, University of Ghana, Legon, Accra, Ghana; 3 Noguchi Memorial Institute for Medical Research, College of Health Sciences, University of Ghana, Legon, Accra, Ghana; 4 Université Jean Lorougnon Guede, Daloa, Côte d’Ivoire; 5 Centre Suisse de Recherches Scientifiques en Côte d’Ivoire, Adiopodume, Côte d’Ivoire; London School of Hygiene and Tropical Medicine, UNITED KINGDOM OF GREAT BRITAIN AND NORTHERN IRELAND

## Abstract

Buruli ulcer (BU), caused by *Mycobacterium ulcerans*, is a neglected tropical skin disease highly endemic in West Africa and Australia. The molecular diversity of *M. ulcerans* strains varies geographically within endemic regions and this directly influences the virulence of mycolactone, the lipid toxin produced by the bacterium which is responsible for disease pathogenesis. This study investigated differences in BU presentation based on various sociodemographic determinants of infection between Ghana and Côte d’Ivoire, two of the most affected countries globally. While the general epidemiology and presentation of confirmed Buruli ulcer cases were similar in both countries, distinct differences emerged in clinical manifestation, perceived transmission routes, and health-seeking behaviours. Interestingly, children in Ghana were the least affected, in direct contrast with Côte d’Ivoire where adults over 60 years had the lowest incidence. In both countries, ulcerative lesions were predominant, typically appearing on the lower limbs. A key distinction was the recall of initial symptoms: most affected individuals in Côte d’Ivoire could not remember how their lesions began, whereas the majority in Ghana reported onset as a boil or oedema or after an injury or bruise from an infected object. These crucial observations are associated with treatment seeking behaviours and have significant implications for early case diagnosis and efforts to understand disease epidemiology.

## Author summary

Buruli ulcer (BU), a debilitating skin disease caused by *Mycobacterium ulcerans*, poses a significant health challenge in West Africa. This study delves into the characteristics of how this disease manifests and is perceived in Ghana and Côte d’Ivoire, two of the most heavily impacted nations. While the overall picture of BU cases is similar in both countries, interesting differences emerged in how the disease presents clinically, how people believe they contracted it, and how they sought treatment. Children in Ghana were less affected compared to Côte d’Ivoire, where adults over 60 showed the lowest incidence. In both countries, ulcers on the lower limbs were the most common type of lesion. A key finding was the difference in how people recollected the start of their symptoms: most in Côte d’Ivoire could not pinpoint the beginning of their lesions, whereas in Ghana, many recalled it starting as a boil, swelling, or after an injury. These distinctions are important because they may directly influence when and how people seek treatment, with significant implications for early diagnosis and disease surveillance strategies.

## Introduction

Buruli ulcer (BU) is a neglected tropical skin disease caused by the bacterium, *Mycobacterium ulcerans* [1,2]. It generally starts as a painless nodule, papule, plaque, small ulcer or diffuse swelling (oedema), which if left untreated can lead to severe ulceration [3] with irregular borders [4]. On few occasions, osteomyelitis as a secondary complication, can occur when treatment is delayed [5]. *M. ulcerans* primarily exhibits its pathogenic effect in the extracellular matrix of subcutaneous tissues of the affected individual [2,6]. Mycolactone, a naturally produced plasmid-encoded polyketide-derived macrolide toxin (pMUM001) within *M. ulcerans*, is the main virulent factor [7]. It suppresses the host’s immune factors from eliciting inflammatory responses and is cytotoxic to most cells including nerve cells making the disease generally painless [8,9].

Buruli ulcer has been reported in 33 countries worldwide with Australia and some West African countries including Ivory Coast, Ghana and Benin reporting the highest number of cases [3]. The latter 3 countries account for more than 70% of globally reported cases [3] and occur in rural areas with inadequate access to good medical care. There has been a gradual decrease in cases reported in the African region in recent years, from 5871 in 2004, 1917 in 2014, and to 1573 cases in 2023 [10], and this may be attributed to under-reporting or under-diagnosis of cases. The disease affects people of all ages and sex, however; it has been commonly identified in the vulnerable population; young (<15 years) and aged (>49 years) [4,11].

Buruli ulcer has been associated with areas of slow flowing or stagnant water bodies [12–14], however, the exact route of *M. ulcerans* transmission is still not well understood. Water bodies in these endemic communities have been subjected to environmental changes such as mining, deforestation as well as flooding and these environmental changes may play a role in the environmental niche of the bacteria [15]. The DNA of *M. ulcerans* has been identified in mosquitoes, aquatic insects, soil, water filtrates, and biofilms [16], with only successful culture from an environmental source reported by Portaels *et al* in 2008 [17]. In Australia, Buruli ulcer is characterized as a zoonosis with possum and mosquitoes mainly involved in the transmission of the disease [18]. No record of human-to-human transmission of the disease has been documented and the disease has also not been found to be contagious [19].

The World Health Organization (WHO) recommended diagnosis of BU is by detection of *M. ulcerans* DNA using Polymerase Chain Reaction (PCR) targeting the IS*2404*, insertion sequence present in over 240 copies in the genome and plasmid [16,20]. Treatment of the disease is by combination therapy with Rifampicin (10 mg/kg orally once daily) and clarithromycin (7.5 mg/kg twice daily) for 8 weeks as the first line of drug treatment [21] and surgery in complicated cases.

Epidemiology and disease presentation of BU vary considerably among different geographical location [22]. For instance, in Africa, a higher percentage of those affected are children (48%) compared to Australia (10%) and Japan (19%) [14,23]. Lesions mostly occur on the limbs with 35% on the upper limb, 55% on the lower limbs with 10% on the other parts of the body [3]. The disease severity has been grouped into three different categories I, II and III depending on the size and spread of the ulcer [3,24].

In a previous study, we showed heterogeneity among *M. ulcerans* from different geographical locations in Ghana and Cote d’Ivoire [25] and this could be important for variation in BU presentation and progression. Knowledge of disease severity would facilitate the work of national control programs in early case detection, monitoring and treatment; however, few comparisons have been done on cases between similar geographical locations in Africa. We hypothesized that there would be significant differences in the clinical presentation and epidemiological patterns of Buruli ulcer between Ghana and Côte d’Ivoire, despite their geographic proximity and similar endemic status. To test this, characteristics of confirmed BU cases were compared between both countries with an in-depth evaluation of disease progression patterns, patients’ recall of initial symptoms, and treatment-seeking behaviours.

## Method

### Ethics statement

Ethics approval of this study was sought from the Institutional Review Board of Noguchi Memorial Institute for Medical Research (052/17–18), Ghana Health Service Ethical Review Committee (GHS-ERC017/07/17) as well as Comité National d’Ethique et de la Recherche [26] in Abidjan, Côte d’Ivoire (112–18/MSHP/CNESVS-km). Written informed consent was obtained from all adult participants. For children below 18 years, written informed consent was obtained from parents or legal guardians before enrolling into the study. In cases where participants were unable to read or write, verbal consent was obtained in the presence of a witness and documented.

All participants were informed of their right to withdraw from the study at any time without affecting their access to medical care. Patient confidentiality was maintained throughout the study, and all data were analyzed anonymously to protect participant confidentiality. Additionally, permission and approval was sought from the National Buruli ulcer Program offices, District Health Directorates, and traditional leaders of the respective communities before the commencement of active case search. BU case diagnosis and treatment were provided at no cost to participants.

### Study site

Study sites were selected based on specific criteria to ensure comprehensive representation of BU endemic areas. Selection criteria included: (1) documented BU endemicity with reported cases in previous years, (2) presence of functional health facilities with capacity for BU case identification and management, (3) accessibility for regular research team visits and timely sample transportation to laboratories, and (4) approval and cooperation from health facility staff and district health directorates. All study sites included in this study met all these four criteria.

**Fig 1:**
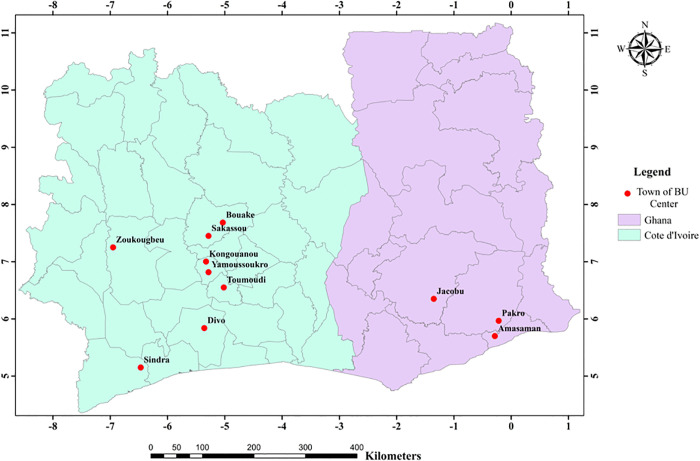
Selected BU endemic sites in Ghana and Côte d’Ivoire for this study. This map was generated using ArcGIS 10.8 software with north arrow, scale bar, and graticule based on the WGS 1984 Geographic Coordinate System, WGS 1984. The data (including base layer and shapefile) that was used, was obtained from the Global Administrative Areas (GADM) database (https://gadm.org). GADM data are freely available for academic use under its license (https://gadm.org/ license.html).

In Ghana, three sites were selected: Amasaman District Hospital in the Ga West Municipal District of Greater Accra, St Peter’s Hospital, Jacobu, [27] in the Amansie Central District of Ashanti region, and Pakro Health Centre in the Akuapim South Municipal District of Eastern region (Fig 1). In Côte d’Ivoire, eight sites were selected: ‘Centre Saint Jean baptiste Vatelot’ in Bouaké, ‘Centre Ub de Divo’ in Divo, ‘Santé Notre Dame du Camel’ in Sakassou, ‘Centre santé de Djenedoufla’ in Sinfra, ‘Centre Saint Michel de Zoukougbeu’ in Zoukougbeu, ‘Centre UB de Kongouanou’ in Kongouanou, ‘Centre de Sainte-famille in Yammousoukro’ and ‘Centre UB de Djekanou’ in Toumoudi (Djekanou) (Fig 1). All the selected study sites are recognized BU endemic areas by their respective national BU control programs.

### Study population

Individuals were eligible for inclusion if they presented with skin lesions clinically suspected to be BU (including nodules, plaques, oedema, or ulcers), resided in or had history of exposure to known BU endemic areas in the selected districts, provided informed consent (or parental/guardian consent for children under 18 years), and were able to provide adequate clinical specimens for laboratory confirmation. Individuals were excluded if they had skin lesions clinically incompatible with BU (e.g., burns, traumatic wounds), refused consent to study participation, or were unable to provide required demographic and clinical information. All participants, regardless of age, gender, occupation, or disease stage, were eligible for inclusion.

### Study design

This community-based, cross-sectional study was conducted between December 2017 and December 2019, allowing simultaneous assessment and comparison of disease prevalence, clinical presentations, and epidemiological patterns across multiple sites in two endemic countries using standardized protocols [10,18,28].

Sample size was calculated based on published BU prevalence data from endemic districts in either country. Using a reported prevalence of 151 per 100,000 inhabitants (P = 0.0015) from an endemic district [29] with 95% confidence level (Z = 1.96) and 5% margin of error (E = 0.05), the minimum sample size was calculated using the formula: n = Z²P(1-P)/E². This calculation yielded a minimum of approximately 2 cases. However, this minimum was clearly insufficient for meaningful comparative analysis between two countries and for robust statistical inference.

Given the known high endemicity of BU in both Ghana and Côte d’Ivoire (ranked among the most affected countries globally), we adopted a consecutive sampling approach. Rather than targeting a pre-specified sample size, all suspected BU cases identified through passive facility-based surveillance and active community case finding during the study period were consecutively enrolled. This approach is appropriate and widely used for rare disease epidemiological research, where case availability is limited by natural occurrence rather than study design constraints, and it maximizes statistical power by capturing all available cases during a defined time frame.

Workshops and trainings were organized in the selected study sites to train health workers on the identification, sample collection, storage and wound management of all suspected BU cases. In addition, permission and approval was sought from the National Buruli ulcer Program offices, District Health Directorates and the traditional leaders of the respective communities before the commencement of active case searches.

### Sample collection

Suspected BU cases were identified using two approaches. Passive case searches were conducted in all selected health facilities where individuals with skin lesions clinically suspected to be BU were enrolled. Active case searches were conducted in individual communities where trained health workers performed outreach activities to identify unreported suspected BU cases. Sterile cotton swabs (for ulcerative lesions) and fine needle aspirates (for pre-ulcerative lesions) were taken by medical practitioners and other trained health workers in the different communities. Three specimens were collected per lesion into sterile screwcap tubes; two in 1ml of 1x Phosphate Buffered Saline (for staining and PCR) and one in PANTA transport media (for culturing). The samples were kept at 4°C in a refrigerator at the respective health centres until ready for further processing and analysis. All participants who tested positive but were not on treatment were directed to the nearest health centres for BU treatment.

### Confirmation of suspected BU cases

All samples were first tested for mycobacteria by acid-fast staining using Ziehl-Neelson’s technique. DNA was then extracted from all the samples using the Zymo Research Quick-DNA Miniprep kit according to Manufacturer’s outlined procedure and stored at -20°C until further use. All samples were then screened via conventional PCR for the presence of mycobacteria by the amplification of 16S rRNA hypervariable region, IS*2404* and IS2606 for *M. ulcerans* DNA; and further for the confirmation of mycolactone producing mycobacteria (MPM) by the amplification of the gene for the Enoyl reductase (ER) domain of the mycolactone producing plasmid (Table 1). All samples that were positive for IS*2404* were considered positive for BU. AFB microscopy was included as part of the diagnostic protocol as recommended by WHO. However, we acknowledge its low sensitivity and specificity, and PCR targeting IS*2404* served as the definitive diagnostic criterion for case confirmation.

**Table 1:** Primers used for mycobacteria identification and Buruli ulcer confirmation.

Primers	Forward (5’ - 3’)	Reverse (5’ - 3’)	Annealing Temp (°C)	size (Bp)
***IS2404***	AGCGACCCCAGTGGATTGGT	CGGTGATCAAGCGTTCACGA	64	492
**IS*2606***	AGGGCAGCGCGGTGATACGG	CAGTGGATTGGTGCCGATCGAG	64	310
**16rRNA**	AAAAAGCGACAAACCTACGAG	AGAGTTTGATCCTGGCTCAG	56	600
**ER**	GAGATCGGTCCCGACGTCTAC	GGCTTGACTGTCACGTAAG	63	730

### Questionnaire and data collection

The questionnaire administered consisted of parameters such as location, gender, age at diagnosis, lesion presentation (nodule, plaque, oedema or ulcer), lesion category (category I: small lesions <5 cm and nodule, category II: lesion size 5–15 cm and oedema, category III lesion size >15 cm as well as multiple lesions), site of lesion, duration of BU lesion as well as treatment options sought by suspected BU cases (hospital, self-care and traditional). The data obtained and lesions identified were reviewed with the help of medical practitioners and other health workers involved in the identification and treatment of neglected tropical skin diseases.

### Statistical analysis

Descriptive analysis was done to present the demographic characteristics of the respondents. The data obtained from each suspected case was recorded on the BU report form (BU 01), and the questionnaire developed for this study. The data was then recorded on Microsoft Excel (Microsoft Office Professional Plus 2016) and analyzed using Excel (Microsoft Office Professional Plus 2016), and Python statistical packages (Python Software Foundation. Python Language Reference, version 3.9 available at http://www.python.org). Graph pad prism 8.4.3, STATA software, version 15.1 (Stata Corp, LP, College Station, USA) was used to develop the graphs.

For categorical variables Pearson’s chi-square test and Fisher’s exact test were used to compare groups [30]. Fisher’s exact test was specifically employed when cell counts were small (<5), as recommended for ensuring validity of results [31]. Medians rather than means were reported for continuous variables [30]. Multinomial logistic regression was used to assess associations between categorical exposures (gender, age groups) and categorical outcomes (lesion types, categories) with multiple levels. This is the appropriate regression method for polytomous dependent variables [32]. For effect measures relative risk ratios with 95% confidence intervals were reported, which are appropriate measures of association for cross-sectional studies comparing groups [33]. We used a significance level of p < 0.05, which is the standard threshold. Variables with p < 0.25 in univariate analysis were included in multivariable models, which is a commonly accepted threshold to avoid excluding potentially important variables [32].

### AI tools and technologies

The human 3D model in Figs 5 and 6 was created using http://openclipart.org from the prompt “[3D shaded human]”.

## Results

### Buruli ulcer cases identified in both countries

A total of three hundred and eighty-two (382) suspected BU cases were recruited into this study from Ghana and Côte d’Ivoire between a two-year span from December 2017 to December 2019. Out of the suspected cases from Ghana, sixty-three (63), twenty-six (26) and sixty-three (63) suspected cases were sampled in Amansie Central District (Ashanti Region), Ga -West Municipal District (Greater Accra region) and Akuapim South Municipal District (Eastern region) respectively (Tables 2 and S1). For Côte d’Ivoire, suspected BU samples were collected from Bouaké (34), Divo (5), Kongouanou (36), Sakassou (18), Sinfra (3), Toumoudi (48), Yammoussoukro (31) and Zoukougbeu (55).

**Table 2:** Positivity rate of suspected BU cases using different mycobacterial confirmatory tools.

Location	IS*2404* n (%)	IS2606 n (%)	ER n (%)	Acid fast n (%)	16S n (%)	Total samples analyzed n (%)	Positivity rate based on IS*2404* (%)
**Ghana (total)**	**104 (68)**	**54 (36)**	**35 (23)**	**5 (3)**	**119 (78)**	**152**	**68**
Ga West Municipal	16 (61)	12 (46)	1 (4)	0	20 (77)	26	62
Akuapim South Municipal	39 (62)	21 (33)	19 (30)	1 (2)	44 (67)	63	62
Amansie Central	49 (78)	21 (33)	15 (24)	4 (6)	55 (87)	63	78
Côte d’Ivoire	196 (85)	169 (73)	151 (66)	11 (5)	220 (96)	230	85
Bouaké	34 (100)	24 (71)	24 (71)	0	34 (100)	34	100
**Divo**	**5 (100)**	**5 (100)**	**5 (100)**	**0**	**5 (100)**	**5**	**100**
Kongouanou	34 (94)	34 (94)	28 (78)	0	36 (100)	36	94
Sakassou	12 (66)	13 (72)	10 (56)	3 (17)	17 (94)	18	67
Sinfra	3 (100)	3 (100)	3 (100)	3 (100)	3 (100)	3	100
Toumoudi/Djekanou	41 (85)	33 (69)	32 (67)	2 (4)	43 (90)	48	85
Yammooussoukro	18 (58)	16 (52)	14 (45)	3 (10)	25 (81)	31	58
Zoukougbeu	49 (89)	39 (71)	35 (64)	0	53 (96)	55	89
**Grand Total**	**300 (78)**	**221 (58)**	**186 (49)**	**16 (4)**	**335 (88)**	**382**	**79**

Only 11/230 (5%) of the samples from Côte d’Ivoire and 5/152 (10%) from Ghana were positive for acid-fast bacilli whereas 335/382 (88%) were positive for mycobacterial infection via PCR amplification of the 16S rRNA hypervariable region. Out of the 382 suspected BU cases, 300 (78%) tested positive for IS*2404*. All epidemiological and clinical analyses presented hereafter are restricted to these 300 IS*2404* positive cases, unless otherwise stated. A total of 221 (58%) samples tested positive for IS*2606* and 186 (49%) for ER respectively (Table 2).

Thus, Ghana, out of the 104/152 (68.4), positive BU cases 49/63 (78%) were from Amansie Central District, 16/26 (61%) from Ga West Municipal District and 39/63 (62%) from Akuapim South District respectively. A higher BU positivity rate was observed in Côte d’Ivoire 196/230 (85%) with 34/34 (100%) in Bouaké, 5/5 (100%) in Divo, 34/36 (94%) in Kongouanou, 12/18 (66%) in Sakassou, 3/3 (100%) in Sinfra, 41/48 (85%) in Toumoudi, 18/37 (58%) in Yamoussoukro and 49/55 (89%) in Zoukougbeu respectively (Table 2).

### Age and gender distribution of Buruli ulcer positive cases

**Fig 2:**
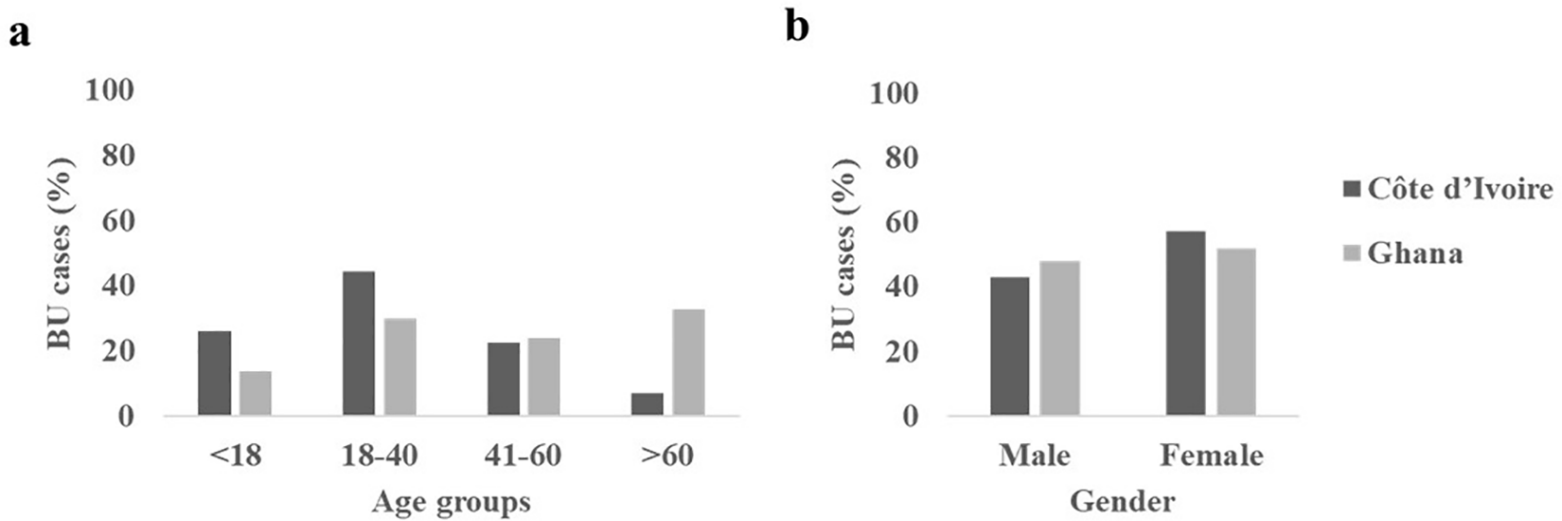
Distribution of Buruli ulcer positive cases in Ghana and Côte d’Ivoire. **a.** Age categories; **b.** Gender distribution.

The ages were categorized into four groups: children under 18 years, young adults (18–40 years), middle-aged adult s(41–60 years) and adults over 60 years (Fig 2a and S2 Table). In general, Côte d’Ivoire had a higher percentage of cases among children under 15 years (38/196; 19.2%) compared to Ghana (10/104; 9.5%), while Ghana had a markedly greater proportion of cases in individuals over 60 years (34/104; 32.4%) compared to Côte d’Ivoire (9/196; 4.5%) (χ² = 44.75, df = 2, p < 0.0001). In Côte d’Ivoire, of the 196 positive cases, 51 (26%) were children under 18 years, 87 (44%) were young adults, 44 (22%) were middle-aged adult and 14 (7%) were above 60 years. The distribution of BU cases across different age groups was not statistically significant in either country (S2 Table) [Fisher exact test, p = 0.179 (Côte d’Ivoire), p = 0.256 (Ghana)].

In Ghana, there was an almost equal gender distribution among those who tested positive for BU; 54/104 (52%) females and 50/104 (48%) males (Figs 2b and S1a). The difference observed was not statistically significant when tested by Pearson’s chi-square test (p = 0.619). A different situation was observed in Côte d’Ivoire, where more males were positive; 112/196 (57%) compared to females, 84/196 (43%) albeit with no statistical significance (Pearson’s chi-square test, p = 0.891) (Figs 2b and S2a). The differences observed in gender between the two countries were also not statistically significant (Chi-square with Yates’ correction, p = 0.257).

### Employment status and educational level of Buruli ulcer positive cases

**Fig 3:**
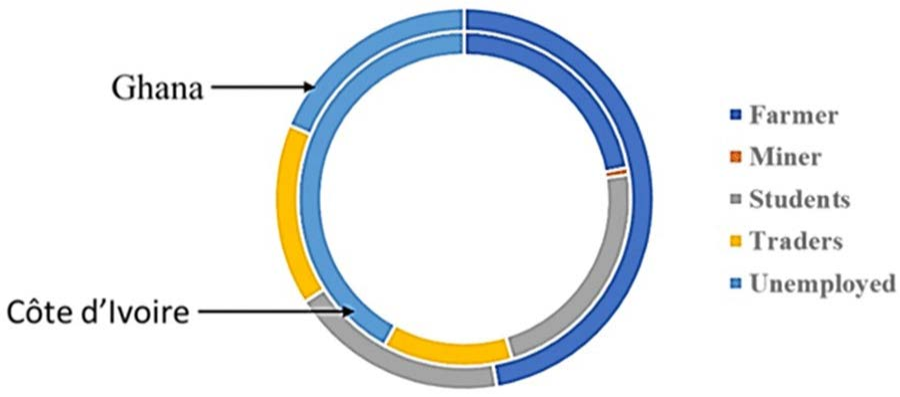
Occupation of participants. Ghana (outer circle); Côte d’Ivoire (inner circle).

The employment status of BU cases was grouped into 6 main categories; farming, students, traders, miners, unemployed and others to determine if occupation was associated with the disease (Fig 3). A higher percentage of farmers were observed in Ghana compared to Côte d’Ivoire. This was followed by student/pupil for both Ghana and Côte d’Ivoire. Even though BU has been associated with areas where the land has been disturbed with activities such as mining, flooding and deforestation, we identified only 1 miner from Côte d’Ivoire and none in the Ghanaian community. Nevertheless, occupation was not found to be significantly associated with BU in both countries [chi-square (fisher exact test); p = 0.367 (Côte d’Ivoire) and p = 0.523 (Ghana)] (S2 Table).

Majority of the participants in Côte d’Ivoire had no formal education compared to Ghana. A larger proportion of those in Ghana had primary education compared to Côte d’Ivoire, whiles fewer had attained a secondary school qualification in both Ghana and Côte d’Ivoire. Difference in the level of education was not found to be statistically significant [Pearson’s chi- square, p = 0.422 (Côte d’Ivoire); p = 0.51 (Ghana)].

### Lesion presentation among Buruli ulcer positive cases

All the different clinical presentations and categories of BU (except papule) were observed in both countries. The location of lesions was grouped into the upper (regions above the waist) and lower parts (regions below the waist) of the body. A total of 242 (80%) lesions were found on the lower part and 56 (19%) in the upper part with 2 (1%) BU cases in Cote d’Ivoire being on both the upper and lower part of the same person.

**Fig 4:**
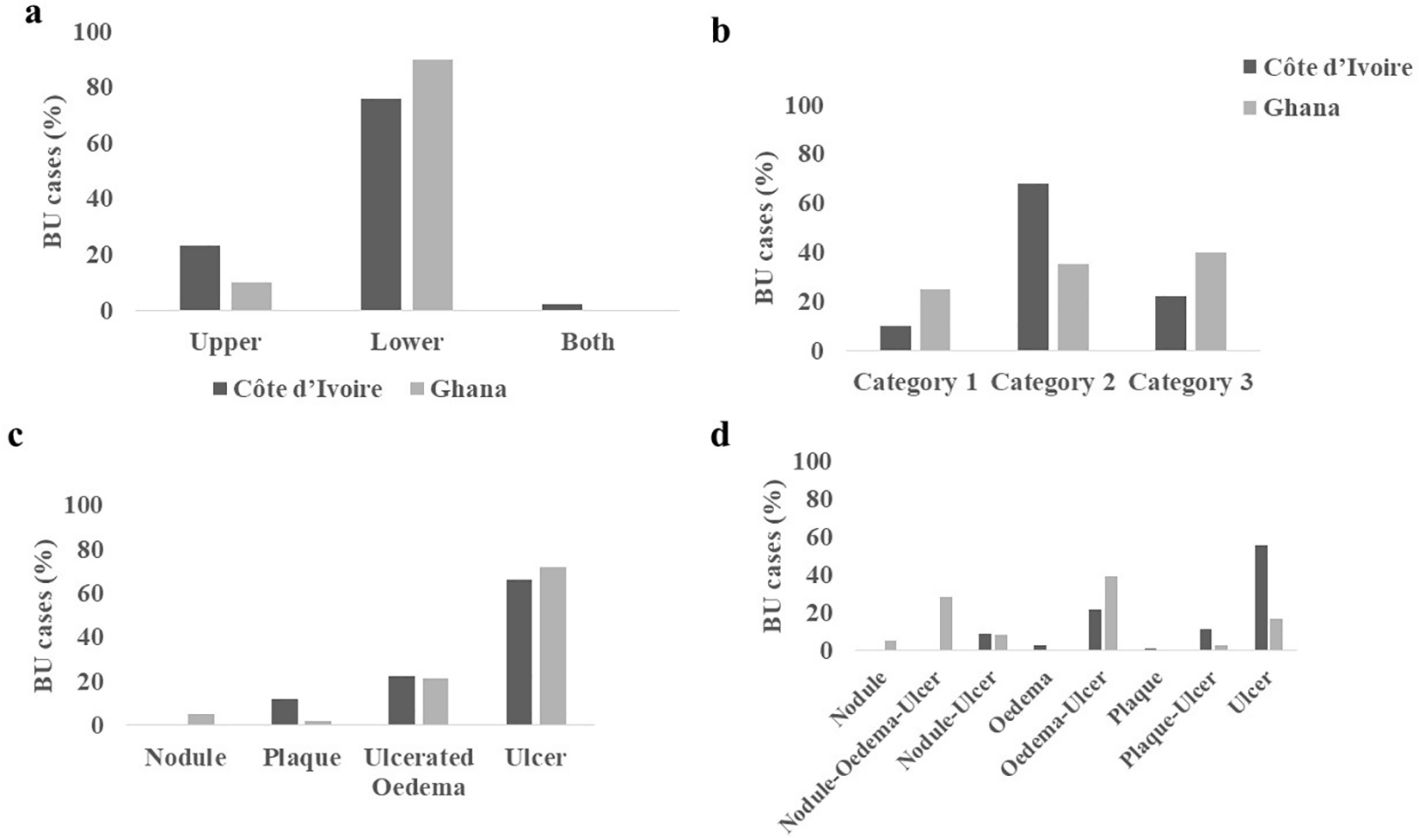
Lesion Presentation among Buruli ulcer positive cases. **a.** Location of BU lesions; b. proportion of category of lesions; c. presentation of lesions; d. progression of lesions.

In Ghana, 94 (90%) were on the lower part of the body (foot, 21 (22%); leg, 72 (77%); 1, (1%) thigh) whiles 10 (10%) were on the upper part of the body (shoulders (trunk), 2 (20%); cheek, 1 (10%), arm 7/10 (70%) (Figs 4a and S1b). In Côte d’Ivoire, 148/196 (76%) of the lesions were located on the lower part (ankle/foot, 105 (71%); leg, 30 (20%); genitals, 3 (2%); thigh, 10 (7%)), 46/196 (23%) were on the upper part of the body (arm, 36 (79%); Cheek, 2 (4%); trunk, 8 (17%)) and 2/196 (1%) were on both parts of the same person (Figs 4a and S2b). Differences in the location of the lesions between the two countries were statistically significant (Fisher exact test, p = 0.007).

All three categories of BU lesions were observed in this study. A total of 44 (15%) were category I, 169 (56%) in category II and 84 (28%) in category III (Fig 4b). In Ghana category III lesions were the most observed, followed by category II and the least being category I affected persons respectively (S1c Fig), however, this observation was not statistically significant (Fisher exact test, p = 0.066. The trend was different in Côte d’ Ivoire where the highest BU category was category II, followed by category III and the least being category I (S2c Fig). Differences in disease categories in Côte d’ Ivoire was not found to be statistically significant (Fisher exact test, p = 0.404).

This study identified a total of 204 (68%) lesions as ulcers, 66 (22%) were ulcerated oedema, 5 (2%) were nodules and 25 (8%) were plaques (Figs 4c, S1d and S2d). Majority of the lesions observed were ulcers in both Ghana and Côte d’Ivoire. This was followed by ulcerated oedema, whiles nodules were seen only in Ghana. Fewer plaques were observed in Ghana compared to Côte d’Ivoire and the differences in lesion presentations were found to be significant in Côte d’Ivoire but not Ghana [Fisher exact test; p = 0.002 (Côte d’Ivoire); p = 0.918 (Ghana)]. Differences in lesion presentation between the two countries were found to be statistically significant (Fisher exact test with Yates’ correction, p = 0.001) (S2 Table), however, reasons for the significance could not be determined using multinomial logistic regression analysis due to low or absence of some variables in the countries (for example, no nodule in Côte d’Ivoire) (S3 and S4 Tables).

We also sought to determine the different stages of disease presentation and progression. All nodular stage and nodule-oedema-ulcer lesions were identified in Ghana (Fig 4d). Eight (3%) and 17 (6%) lesions that progressed directly from the nodule to ulcer stage were identified in Ghana and in Côte d’Ivoire respectively. Both countries had lesions that had evolved from oedema to ulcer and from plaques to ulcers, however ulcerative lesions were found to be the highest in Côte d’Ivoire.

### Classification of BU lesions based on gender and age

**Fig 5:**
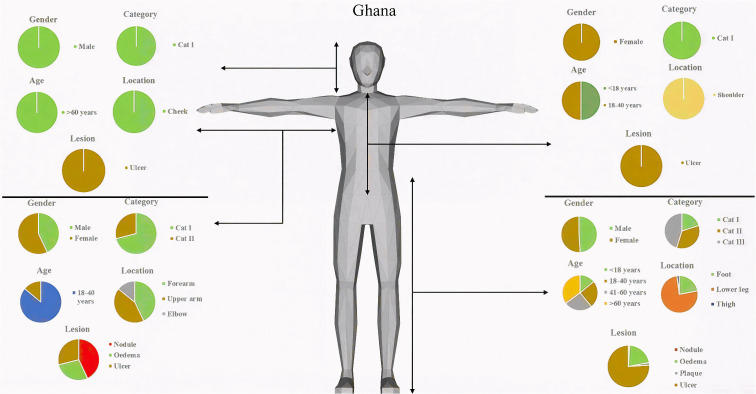
Summary of clinical presentation of BU lesions observed in Ghana. Image generated using http://openclipart.org from the prompt “[3D shaded human]”, openclipart.org, [24-11-2025].

**Fig 6:**
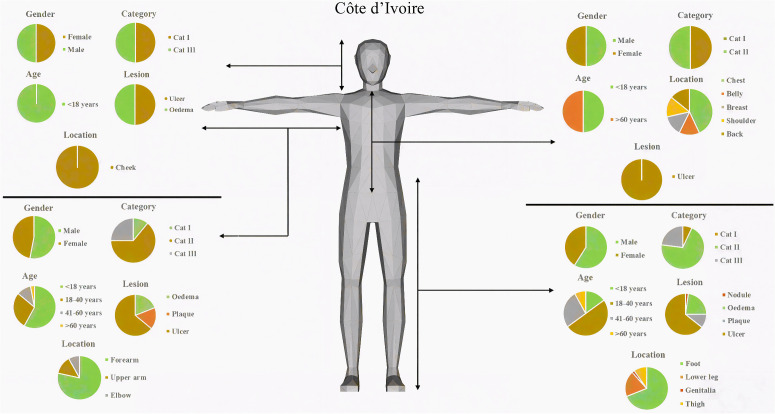
Summary of clinical presentation of BU lesions observed in Côte d’Ivoire. Image generated using http://openclipart.org from the prompt “[3D shaded human]”, openclipart.org, [24-11-2025].

In order to appreciate the diversity in lesion characteristics, we analysed the data in both countries based on gender and age. No nodule was identified among those under 18 years and 41–60 years age group in both countries (Figs 5 and 6). In Ghana, all plaques were observed on adults over 60 years with an even gender distribution, however, for Côte d’Ivoire, majority of the plaques identified were among those under 18 years who were females and both adult groups but none among adults over 60 years.

Children under 18 years with BU lesion in category I were mostly females in Ghana. Category II and category III lesions were identified among all age groups and sexes. In Côte d’ Ivoire, category II and III lesions were identified among all gender and age groups. Category 1 lesions were observed among both genders, but markedly higher in female children under 18 years and none in adults above 60 years.

In Ghana, the lesions on the lower part of the body were evenly distributed on both gender and age groupings. For the upper part of the body, lesions were only found among females below 18 years and the adult groups of both genders with more female presenting (Fig 5). A different trend was observed in Côte d’Ivoire where lesions were distributed equally among both gender and age groups (Fig 6), however unlike Ghana, a higher proportion of lesions identified on the upper part of the body were on children below 18 years.

Lesion presentation was found to be significantly associated with gender in Côte d’Ivoire but not Ghana [Fisher exact test; p = 0.002 (Côte d’Ivoire); p = 0.918 (Ghana)] (S2 Table). Compared to females, males were at a 27% lower risk of getting plaques (Multinomial Regression, relative risk ratio: 27, 95% CI 0.0098 – 0.76, P-value = 0.013), however, no such association was identified in Ghana (S3 and S4 Tables).

No association was found between gender and the category and location of BU lesion in both countries [p = 0.404 (Côte d’Ivoire), p= (0.066 (Ghana)] and [chi-square, p = 0.270 (Côte d’Ivoire), p = 0.591 respectively] (S5 Table). Gender was however found to be associated with BU progression in Côte d’Ivoire (chi-square, p = 0.005), but not in Ghana (chi-square, p = 0.246). Females were at a higher risk (76%) of BU progression from plaque to ulcer ((Multinomial Regression, relative risk ratio: 24, 95% CI 0.067 – 0.865, P-value = 0.029, appendix 8) (S3 and S4 Tables).

### Participants recollection on how lesion began

**Fig 7:**
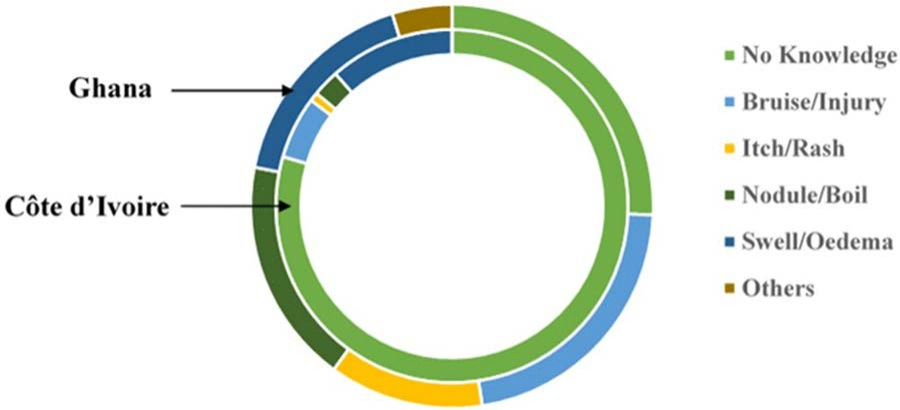
Representation on initiation of BU lesions in both countries. Outer circle (Ghana), inner circle (Côte d’Ivoire).

Given that Buruli ulcer transmission remains unknown, enquiries were made about the possible initiation of infection among the participants. As expected, a large proportion of individuals did not recall how the lesion started. (Fig 7 and S2 Table). Specifically, in Ghana, 24 (23%) respondents indicated they had no knowledge on how the lesion begun, 22 (21%) indicated it started as a boil or nodule, 23 (22%) as a bruise or injury, 15 (15%) as an itch or rash, and 20 (19%) as a swelling. For Côte d’Ivoire, 145 (74%) had no knowledge on how the BU lesion started, 3 (2%) indicated it started as a boil or nodule, 31 (16%) as a bruise or injury, 1 (0.5%) as an itch or rash and 16 (8%) as a swelling.

### Treatment options of Buruli ulcer patients

**Fig 8:**
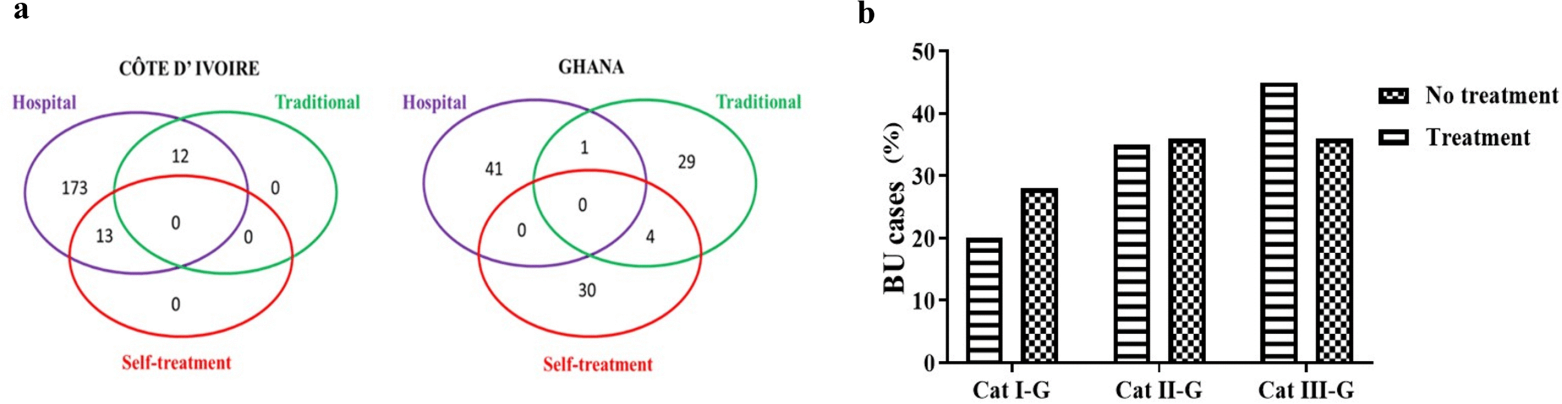
Treatment seeking behaviour of patients. **a.** Different treatment options for the observed BU cases; **b.** Category of lesions among the untreated and treated BU cases in Ghana.

The treatment options were grouped into 3; hospital/clinic, traditional and self-treatment at home based on where participants were enrolled for this study. Passive enrollment was for patients in the hospitals while active case searches were used to enroll patients from homes and in traditional healing facilities. In Ghana hospitals or traditional healer were seen as viable treatment options as compared to Côte d’Ivoire where all patients identified mainly sought treatment from the hospital, with a small number in combination with traditional and self-treatment at home (Fig 8a and S6 Table). Interestingly, out of the 56/104 (54%) BU patients receiving antibiotic treatment in Ghana, only 5/104 (5%) had started the recommended antibiotic treatment (rifampicin and clarithromycin). The rest were on other antibiotics such as amoxiclav and penicillin for treatment of the lesion probably because the cases were yet to be confirmed by PCR. The situation was different in Côte d’Ivoire where all BU cases identified were on the recommended antibiotic treatment (rifampicin and streptomycin). Of note in Ghana was the continuous treatment with other antibiotics after the recommended BU therapy for the prevention of secondary bacterial infection associated with BU and delay in wound healing.

Comparisons between antibiotic treatment and lesion category were also determined in Ghana. It was observed that patients were likely to seek treatment when lesions were in category II or III compared to category I (Fig 8b). This comparison was not done for Côte d’Ivoire since all BU cases sought treatment at the hospital and were on antibiotic treatment.

## Discussion

This comparative study reveals important epidemiological and clinical differences in BU presentation between Ghana and Côte d’Ivoire, two neighboring highly endemic countries. While both countries share similar geography and socioeconomic conditions, our findings demonstrate substantial variation in disease patterns, challenging assumptions about uniform BU epidemiology across West Africa.

Contrary to typical patterns in Africa where BU predominantly affects children [11,34], our study identified distinct age distributions in each country. Young adults were most affected in Côte d’Ivoire, while adults over 60 years showed highest prevalence in Ghana. Conversely, adults over 60 years had the lowest incidence in Côte d’Ivoire. These differences may reflect varying exposure patterns related to occupational activities, domestic responsibilities, or mobility differences across age groups. We found farmers to be the most affected occupational group in both countries, consistent with studies identifying cocoa plantation exposure as a risk factor [15].

The lower incidence among young children and elderly adults could be attributed to limited movement and reduced environmental contact with water bodies where *M. ulcerans* is believed to be present. Gender was also not found to be a risk for the occurrence of BU in both countries and several studies corroborate this finding [34,35]. BU incidence was however higher among female children below 18 years compared to males in the same age category.

A striking finding was the dramatic difference in disease onset recall between countries and this is consistent with the variability in disease incubation period [36]. In Côte d’Ivoire, most patients could not recall how their lesions began, compared to Ghana where most patients recalled specific onset patterns (boil, oedema, injury, or bruise). This difference has important implications for surveillance and early detection strategies. *M. ulcerans* exposure to cuts or bruises has been reported as a risk factor for BU transmission. However, an animal infection study conducted on hairless guinea pigs observed that infection through passive inoculation into an existing abrasion was not sufficient to cause a productive infection [37]. Other studies have suggested the possibility of *M. ulcerans* infection developing at the site of trauma to the skin [38]. This may likely represent the huge proportion of BU affected individuals who reported itchy skin before the development of the ulcer.

In Ghana, community education emphasizing recognition of specific early signs (nodules, boils, injuries) may improve early case detection. In Côte d’Ivoire, surveillance strategies may need to focus on identifying more advanced lesions and reducing delays between symptom onset and healthcare seeking. The reasons for this recall difference require further investigation but may reflect actual differences in symptom characteristics, variations in disease progression rates, differences in healthcare access, or levels of disease awareness.

Significant differences in lesion characteristics were observed between countries. Nodules were identified exclusively in Ghana, while plaques were significantly more common in Côte d’Ivoire. Anatomical distribution also differed, with upper body lesions more common among children in Côte d’Ivoire compared to adults in Ghana. Both countries showed predominance of ulcerative lesions on lower limbs, consistent with global patterns [16,39], and the location on unprotected body parts supports insect mediated or direct inoculation transmission hypotheses. Previous work in our group identified distinct *M. ulcerans* genotype distributions between the two countries, with genotypes A, H, J, and K unique to Côte d’Ivoire and genotypes E and Y unique to Ghana [25]. This genetic diversity could influence virulence, symptom presentation, and disease progression, though this hypothesis requires further investigation.

Treatment-seeking patterns differed substantially between countries. In Côte d’Ivoire, all identified BU patients were treated using the 8-week combination of rifampicin (10mg/kg daily) and streptomycin (15mg/kg intramuscular) and preferred the hospital-stay for better monitoring of disease progression or regression. The current recommendation by WHO supports outpatient care but requires frequent follow-up and wound management systems [40]. In Ghana, many BU patients had not yet commenced the recommended hospital-based treatment, and some were on non-standard antibiotics, likely because lesions had not yet been confirmed as BU by PCR.

The observed treatment seeking behaviour could explain the higher proportion of lesions in Côte d’Ivoire being Category II, while Ghana had more Category III lesions. This suggests a complex interaction between healthcare seeking, disease progression, and diagnostic confirmation and reflects the continuous need for better healthcare access, stronger BU awareness programs and ultimately institution of integrated awareness campaigns for neglected tropical skin diseases in general by national control programs. Early misdiagnosis remains a challenge in both countries, as nodular lesions are often mistaken for lipomas or other subcutaneous infections [3].

Our findings demonstrate that even neighboring endemic countries with similar conditions can exhibit substantial epidemiological differences. This underscores the need for tailored country-specific approaches rather than uniform regional strategies. For Ghana, priorities include strengthening early detection through community education about specific onset symptoms, improving diagnostic confirmation rates, and expanding access to recommended antibiotic treatment. For Côte d’Ivoire, priorities include understanding why symptom recall is poor, maintaining strong treatment access, and potentially focusing on preventing progression to advanced categories. The capacity building from community health worker trainings conducted in this study represents an important foundation for improving awareness and early case detection in both countries.

This study had some limitations. Some BU cases may have been missed because the study enrolled only people who came to health facilities or were reached during active case searches in the community. The study relied on participants to accurately report information about their age, occupation, and how their disease started, which may not always be accurate. Detailed information was not obtained on income levels, water contact, or additional environmental factors that may have better explained peculiar differences observed. Despite these limitations, this study provides important information comparing BU patterns in two countries with high disease rates, which can help improve disease detection and control efforts.

## Conclusion

This study presents a comprehensive comparison of BU epidemiology in Ghana and Côte d’Ivoire with a focus on understanding disparities in disease presentation and demographic patterns. This information is an important step towards understanding possible risk factors of infection based on sociodemographic factors. Similar studies in Australia have contributed to identifying the associations between disease epidemiology and repeated mosquito bites. Inefficiencies exist in relying on patient recall in uncovering the mode of transmission and capacity building inherent in our community trainings on identifying suspicious lesions, is an important approach to inform country-specific early case detection. Point of care rapid diagnostic test kit will be highly beneficial in reducing lesion progression and, continued efforts to understanding BU epidemiology through comparative studies are essential for developing evidence-based prevention and control strategies.

## Supporting information

S1 Table Socio-demographic background of BU positive versus BU negative cases identified in Côte d’Ivoire and Ghana. (DOCX)

S2 Table Lesion presentations on BU cases in Côte d’Ivoire and Ghana by age groups. (DOCX)

S3 Table Multinomial logistic regression analysis for Cote d’Ivoire using sex and age groups as independent variable. (DOCX)

S4 Table Multinomial logistic regression analysis for Ghana using sex and age groups as independent variable. (DOCX)

S5 Table Comparison of BU lesion characteristics, between Côte d’Ivoire and Ghana (Chi-square analysis). (DOCX)

S6 Table Treatment seeking behaviour among the BU cases in Ghana and Côte d’Ivoire by age. (DOCX)

S1 Fig Graphical summary of age and gender distribution with BU case presentation in Ghana. (TIFF)

S2 Fig Graphical summary of age and gender distribution with BU case presentation in Côte d’Ivoire. (TIF)
