# Supplementary material for: Comparative analysis of Buruli ulcer in Ghana and Côte d’Ivoire: A cross-sectional study
Source: PLoS Negl Trop Dis. 2026 Jan 12;20(1):e0013912. doi: 10.1371/journal.pntd.0013912 (PMC12822952; doi:10.1371/journal.pntd.0013912)
Supplement: S1 Table — (DOCX) [file pntd.0013912.s001.docx]

| **Demographics** | **Côte d’Ivoire** | | | |  | **Ghana** | | | |
| --- | --- | --- | --- | --- | --- | --- | --- | --- | --- |
|  | **Negative** | **Positive** | **Total** |  |  | **Negative** | **Positive** | **Total** |  |
|  | **n (%)** | **n (%)** | **n (%)** | **P-value** |  | **n (%)** | **n (%)** | **n (%)** | **P-value** |
| **Age groups (in years)** |  |  |  |  |  |  |  |  |  |
| <18 | 4 (11.8) | 51 (26) | 55 (23.9) | 0.179 |  | 3 (6.3) | 14 (13.5) | 17 (11.2) | 0.256 |
| 18-40 | 18 (52.9) | 87 (44.4) | 105 (45.7) |  |  | 21 (43.8) | 31 (29.8) | 52 (34.2) |  |
| 41-60 | 11 (32.4) | 44 (22.4) | 55 (23.9) |  |  | 12 (25) | 25 (24) | 37 (24.3) |  |
| >60 | 1 (2.9) | 14 (7.1) | 15 (6.5) |  |  | 12 (25) | 34 (32.7) | 46 (30.3) |  |
| Total | 34 (100) | 196 (100) | 230 (100) |  |  | 48 (100) | 104 (100) | 152 (100) |  |
| **Gender** |  |  |  |  |  |  |  |  |  |
| Female | 15 (44.1) | 84 (42.9) | 99 (43) | 0.891 |  | 27 (56.3) | 54 (51.9) | 81 (53.3) | 0.619 |
| Male | 19 (55.9) | 112 (57.1) | 131 (57) |  |  | 21 (43.8) | 50 (48.1) | 71 (46.7) |  |
| Total | 34 (100) | 196 (100) | 230 (100) |  |  | 48 (100) | 104 (100) | 152 (100) |  |
| **Occupation** |  |  |  |  |  |  |  |  |  |
| Farmers | 10 (29.4) | 32 (16.3) | 42 (18.3) | 0.367 |  | 22 (45.8) | 43 (41.3) | 65 (42.8) |  |
| Miners | 0 (0.0) | 1 (0.5) | 1 (0.4) |  |  |  |  |  |  |
| Students | 6 (17.6) | 34 (17.3) | 40 (17.4) |  |  | 4 (8.3) | 17 (16.3) | 21 (13.8) | 0.523 |
| Traders | 5 (14.7) | 19 (9.7) | 24 (10.4) |  |  | 4 (8.3) | 14 (13.5) | 18 (11.8) |  |
| Unemployed | 8 (23.5) | 62 (31.6) | 70 (30.4) |  |  | 10 (20.8) | 16 (15.4) | 26 (17.1) |  |
| Others | 5 (14.7) | 48 (24.5) | 53 (23) |  |  | 8 (16.7) | 14 (13.5) | 22 (14.5) |  |
| Total | 34 (100) | 196 (100) | 230 (100) |  |  | 48 (100) | 104 (100) | 152 (100) |  |
| **Marital Status** |  |  |  |  |  |  |  |  |  |
| Divorce/Separated |  |  |  |  |  | 4 (8.3) | 5 (4.8) | 9 (5.9) | 0.727 |
| Married | 8 (23.5) | 36 (18.4) | 44 (19.1) | 0.693 |  | 21 (43.8) | 49 (47.1) | 70 (46.1) |  |
| Single | 6 (17.6) | 35 (17.9) | 41 (17.8) |  |  | 18 (37.5) | 35 (33.7) | 53 (34.9) |  |
| Widowed | 2 (5.9) | 24 (12.2) | 26 (11.3) |  |  | 5 (10.4) | 15 (14.4) | 20 (13.2) |  |
| Missing | 18 (52.9) | 101 (51.5) | 119 (51.7) |  |  |  |  |  |  |
| Total | 34 (100) | 196 (100) | 230 (100) |  |  | 48 (100) | 104 (100) | 152 (100) |  |
| **Education level** |  |  |  |  |  |  |  |  |  |
| None | 23 (67.6) | 115 (58.7) | 138 (60) | 0.422 |  | 13 (27.1) | 31 (29.8) | 44 (28.9) | 0.51 |
| Primary | 5 (14.7) | 49 (25) | 54 (23.5) |  |  | 24 (50) | 42 (40.4) | 66 (43.4) |  |
| Secondary | 6 (17.6) | 32 (16.3) | 38 (16.5) |  |  | 11 (22.9) | 31 (29.8) | 42 (27.6) |  |
| Total | 34 (100) | 196 (100) | 230 (100) |  |  | 48 (100) | 104 (100) | 152 (100) |  |

S1 Table: Socio-Demographic Background of BU Positive versus BU Negative Cases Identified in Côte d’Ivoire and Ghana

**NB: Statistical significance was determine using Pearson chi-square for large variables and Fisher exact test for variables less than 5**
