# Supplementary material for: Comparative analysis of Buruli ulcer in Ghana and Côte d’Ivoire: A cross-sectional study
Source: PLoS Negl Trop Dis. 2026 Jan 12;20(1):e0013912. doi: 10.1371/journal.pntd.0013912 (PMC12822952; doi:10.1371/journal.pntd.0013912)
Supplement: S2 Table — (DOCX) [file pntd.0013912.s002.docx]

S2 Table: Lesion Presentations on BU Cases in Côte d’Ivoire and Ghana by Age Groups


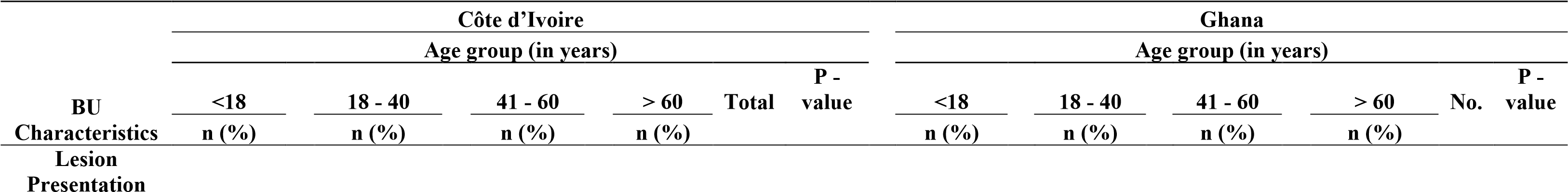


| Nodule |  |  |  |  |  |  |  |  |  |  | 0 (0.0) |  | 3 (75.0) |  | 0 (0.0) |  | 1 (25.0) | 4 |  |
| --- | --- | --- | --- | --- | --- | --- | --- | --- | --- | --- | --- | --- | --- | --- | --- | --- | --- | --- | --- |
| Oedema | 13 (29.5) |  | 18 (40.9) |  | 8 (18.2) |  | 5 (11.4) | 44 |  |  | 2 (9.1) |  | 7 (31.8) |  | 5 (22.7) |  | 8 (36.4) | 22 | 0.383 |
| Plaque | 9 (39.1) |  | 8 (34.8) |  | 6 (26.1) |  | 0 (0.0) | 23 | 0.373 |  | 0 (0.0) |  | 0 (0.0) |  | 0 (0.0) |  | 2 (100) | 2 |  |
| Ulcer | 29 (22.5) |  | 61 (47.3) |  | 30 (23.3) |  | 9 (7.0) | 129 |  |  | 12 (15.8) |  | 21 (27.6) |  | 20 (26.3) |  | 23 (30.3) | 76 |  |
| Total | 51 (26.0) |  | 87 (44.4) |  | 44 (22.4) |  | 14 (7.1) | 196 |  |  | 14 (13.5) |  | 31 (29.8) |  | 25 (24.0) |  | 34 (32.7) | 104 |  |
|  |  |  |  |  |  |  |  |  |  |  |  |  |  |  |  |  |  |  |  |
| **Category** |  |  |  |  |  |  |  |  |  |  |  |  |  |  |  |  |  |  |  |
| I | 6 (31.6) |  | 10 (52.6) |  | 2 (10.5) |  | 1 (5.3) | 19 |  |  | 5 (18.5) |  | 9 (33.3) |  | 4 (14.8) |  | 9 (33.3) | 27 |  |
| II | 37 (27.8) |  | 56 (42.1) |  | 31 (23.3) |  | 9 (6.8) | 133 | 0.715 |  | 2 (5.7) |  | 11 (31.4) |  | 11 (31.4) |  | 11 (31.4) | 35 | 0.609 |
| III | 8 (18.2) |  | 21 (47.7) |  | 11 (25.0) |  | 4 (9.1) | 44 |  |  | 7 (16.7) |  | 11 (26.2) |  | 10 (23.8) |  | 14 (33.3) | 42 |  |
| Total | 51 (26.0) |  | 87 (44.4) |  | 44 (22.4) |  | 14 (7.1) | 196 |  |  | 14 (13.5) |  | 31 (29.8) |  | 25 (24.0) |  | 34 (32.7) | 104 |  |
|  |  |  |  |  |  |  |  |  |  |  |  |  |  |  |  |  |  |  |  |
| **Lesion location** |  |  |  |  |  |  |  |  |  |  |  |  |  |  |  |  |  |  |  |
| Arm | 21 (58.3) |  | 10 (27.8) |  | 4 (11.1) |  | 1 (2.8) | 36 |  |  | 0 (0.0) |  | 6 (85.7) |  | 1 (14.3) |  | 0 (0.0) | 7 |  |
| Cheek | 2 (100) |  | 0 (0.0) |  | 0 (0.0) |  | 0 (0.0) | 2 |  |  | 0 (0.0) |  | 0 (0.0) |  | 0 (0.0) |  | 1 (100) | 1 |  |
| Foot | 10 (9.5) |  | 55 (52.4) |  | 32 (30.5) |  | 8 (7.6) | 105 |  |  | 2 (9.5) |  | 6 (28.6) |  | 6 (28.6) |  | 7 (33.3) | 21 | 0.154 |
| Genitalia | 2 (66.7) |  | 1 (33.3) |  | 0 (0.0) |  | 0 (0.0) | 3 | <0.001 |  |  |  |  |  |  |  |  |  |  |
| Leg | 8 (26.7) |  | 14 (46.7) |  | 5 (16.7) |  | 3 (10.0) | 30 |  |  | 11 (15.3) |  | 17 (23.6) |  | 18 (25) |  | 26 (36.1) | 72 |  |
| Thigh | 3 (30.0) |  | 4 (40.0) |  | 3 (30.0) |  | 0 (0.0) | 10 |  |  | 0 (0.0) |  | 1 (100) |  | 0 (0.0) |  | 0 (0.0) | 1 |  |

| Trunk | 5 (62.5) |  | 2 (25.0) |  | 0 (0.0) |  | 1 (12.5) | 8 |  |  | 1 (50.0) |  | 1 (50.0) |  | 0 (0.0) |  | 0 (0.0) | 2 |  |
| --- | --- | --- | --- | --- | --- | --- | --- | --- | --- | --- | --- | --- | --- | --- | --- | --- | --- | --- | --- |
| Whole body | 0 (0.0) |  | 1 (50.0) |  | 0 (0.0) |  | 1 (50.0) | 2 |  |  |  |  |  |  |  |  |  |  |  |
| Total | 51 (26) |  | 87 (44.4) |  | 44 (22.4) |  | 14 (7.1) | 196 |  |  | 14 (13.5) |  | 31 (29.8) |  | 25 (24) |  | 34 (32.7) | 104 |  |
|  |  |  |  |  |  |  |  |  |  |  |  |  |  |  |  |  |  |  |  |
| **Lesion location** |  |  |  |  |  |  |  |  |  |  |  |  |  |  |  |  |  |  |  |
| Both | 0 (0.0) |  | 1 (50.0) |  | 0 (0.0) |  | 1 (50.0) | 2 |  |  |  |  |  |  |  |  |  |  |  |
| Lower part | 23 (15.5) |  | 74 (50.0) |  | 40 (27.0) |  | 11 (7.4) | 148 | <0.001 |  | 13 (13.8) |  | 24 (25.5) |  | 24 (25.5) |  | 33 (35.1) | 94 |  |
| Upper part | 28 (60.9) |  | 12 (26.1) |  | 4 (8.7) |  | 2 (4.3) | 46 |  |  | 1 (10.0) |  | 7 (70.0) |  | 1 (10.0) |  | 1 (10.0) | 10 | 0.033 |
| Total | 51 (26.0) |  | 87 (44.4) |  | 44 (22.4) |  | 14 (7.1) | 196 |  |  | 14 (13.5) |  | 31 (29.8) |  | 25 (24) |  | 34 (32.7) | 104 |  |
|  |  |  |  |  |  |  |  |  |  |  |  |  |  |  |  |  |  |  |  |
| **Stage** |  |  |  |  |  |  |  |  |  |  |  |  |  |  |  |  |  |  |  |
| Nodule-Ulcer | 5 (29.4) |  | 8 (47.1) |  | 3 (17.6) |  | 1 (5.9) | 17 |  |  | 0 (0) |  | 3 (60) |  | 1 (20.0) |  | 1 (20.0) | 5 |  |
| Oedema | 1 (20.0) |  | 3 (60.0) |  | 1 (20.0) |  | 0 (0.0) | 5 |  |  | 2 (6.9) |  | 12 (41.4) |  | 7 (24.1) |  | 8 (27.6) | 29 |  |
| Oedema-Ulcer | 15 (35.7) |  | 15 (35.7) |  | 7 (16.7) |  | 5 (11.9) | 42 |  |  | 2 (25) |  | 3 (37.5) |  | 2 (25.0) |  | 1 (12.5) | 8 |  |
| Plaque | 0 (0.0) |  | 1 (100) |  | 0 (0.0) |  | 0 (0.0) | 1 | 0.56 |  | 7 (17.1) |  | 10 (24.4) |  | 10 (24.4) |  | 14 (34.1) | 41 | 0.582 |
| Plaque-Ulcer | 9 (40.9) |  | 7 (31.8) |  | 6 (27.3) |  | 0 (0.0) | 22 |  |  | 1 (33.3) |  | 0 (0.0) |  | 0 (0.0) |  | 2 (66.7) | 3 |  |
| Ulcer | 21 (19.3) |  | 53 (48.6) |  | 27 (24.8) |  | 8 (7.3) | 109 |  |  | 2 (11.1) |  | 3 (16.7) |  | 5 (27.8) |  | 8 (44.4) | 18 |  |
| Total | 51 (26) |  | 87 (44.4) |  | 44 (22.4) |  | 14 (7.1) | 196 |  |  | 14 (13.5) |  | 31 (29.8) |  | 25 (24) |  | 34 (32.7) | 104 |  |
|  |  |  |  |  |  |  |  |  |  |  |  |  |  |  |  |  |  |  |  |
| **Possible Initiation of lesion** | |  |  |  |  |  |  |  |  |  |  |  |  |  |  |  |  |  |  |
| Boil/Nodule 0 (0.0) | |  | 2 (66.7) |  | 1 (33.3) |  | 0 (0.0) | 3 |  |  | 3 (13.6) |  | 8 (36.4) |  | 5 (22.7) |  | 6 (27.3) | 22 |  |
| Bruise/Injury 9 (29.0) | |  | 13 (41.9) |  | 8 (25.8) |  | 1 (3.2) | 31 |  |  | 5 (21.7) |  | 7 (30.4) |  | 5 (21.7) |  | 6 (26.1) | 23 |  |
| Itch/Rush 0 (0.0) | |  | 0 (0.0) |  | 1 (100) |  | 0 (0.0) | 1 |  |  | 1 (6.7) |  | 4 (26.7) |  | 3 (20.0) |  | 7 (46.7) | 15 |  |
| Swells/Oedema 7 (43.8) | |  | 4 (25.0) |  | 4 (25.0) |  | 1 (6.3) | 16 |  |  | 2 (10.0) |  | 4 (20.0) |  | 7 (35.0) |  | 7 (35) | 20 |  |
| Dont't Know 35 (24.1) | |  | 68 (46.9) |  | 30 (20.7) |  | 12 (8.3) | 145 | <0.001 |  | 3 (12.5) |  | 8 (33.3) |  | 5 (20.8) |  | 8 (33.3) | 24 | 0.93 |
| Total 51 (26.0) | |  | 87 (44.4) |  | 44 (22.4) |  | 14 (7.1) | 196 |  |  | 14 (13.5) |  | 31 (29.8) |  | 25 (24) |  | 34 (32.7) | 104 |  |

**NB:** Statistical significance was determined using Pearson chi-square for large samples size and Fisher exact test for sample size less than 5.
