# Supplementary material for: Comparative analysis of Buruli ulcer in Ghana and Côte d’Ivoire: A cross-sectional study
Source: PLoS Negl Trop Dis. 2026 Jan 12;20(1):e0013912. doi: 10.1371/journal.pntd.0013912 (PMC12822952; doi:10.1371/journal.pntd.0013912)
Supplement: S4 Table — (DOCX) [file pntd.0013912.s004.docx]

**S4 Table: Multinomial Logistic Regression Analysis for Ghana Using Sex and Age groups as Independent Variable**

| **Variables** | **Exponentiated coefficient (b)** | **Standard. Errors** | **z-Score** | **P>z** | **[95% Confidence interval) Lower** | **[95% Confidence interval) Upper** |
| --- | --- | --- | --- | --- | --- | --- |
|  |  |  |  |  |  |  |
| **Nodule** | **(base outcome)** |  |  |  |  |  |
|  |  |  |  |  |  |  |
| **Oedema** |  |  |  |  |  |  |
| **sex** |  |  |  |  |  |  |
| **Female** | 1 |  |  |  |  |  |
| **Male** | 0.6182222 | 0.6182319 | -0.48 | 0.631 | 0.0870823 | 4.388939 |
|  |  |  |  |  |  |  |
| **Age group (in years)** | |  |  |  |  |  |
| **18-40** | 1.804509 | 2.5067 | 0.42 | 0.671 | 0.1185573 | 27.46564 |
| **41-60** | 6057854 | 9.25E+09 | 0.01 | 0.992 | 0 | . |
| **>60** | 3.948783 | 6.404041 | 0.85 | 0.397 | 0.164439 | 94.8247 |
|  |  |  |  |  |  |  |
| _cons | 2.625981 | 3.58947 | 0.71 | 0.48 | 0.1802099 | 38.26523 |
|  |  |  |  |  |  |  |
| **Plaque** |  |  |  |  |  |  |
| **sex** |  |  |  |  |  |  |
| **Female** | 1 |  |  |  |  |  |
| **Male** | 0.2876637 | 0.4475509 | -0.8 | 0.423 | 0.0136325 | 6.070093 |
|  |  |  |  |  |  |  |
| **Age category** |  |  |  |  |  |  |
| **<18** | 1 |  |  |  |  |  |
| **18-40** | 0.9854816 | 3332.874 | 0 | 1 | 0 | . |
| **41-60** | 2.23E+13 | 7.37E+16 | 0.01 | 0.993 | 0 | . |
| **>60** | 1.67E+07 | 4.91E+10 | 0.01 | 0.995 | 0 | . |
|  |  |  |  |  |  |  |
| _cons | 2.09E-07 | 0.000613 | -0.01 | 0.996 | 0 | . |
|  |  |  |  |  |  |  |
| **Ulcer** |  |  |  |  |  |  |
| **sex** |  |  |  |  |  |  |
| **Female** | 1 |  |  |  |  |  |
| **Male** | 0.5012538 | 0.4715971 | -0.73 | 0.463 | 0.0792898 | 3.168822 |
|  |  |  |  |  |  |  |
| **Age group (in years)** | |  |  |  |  |  |
| **<18** | 1 |  |  |  |  |  |
| **18-40** | 0.8843298 | 1.061683 | -0.1 | 0.918 | 0.084082 | 9.300906 |
| **41-60** | 5459222 | 8.34E+09 | 0.01 | 0.992 | 0 | . |
| **>60** | 2.453936 | 3.564575 | 0.62 | 0.537 | 0.142368 | 42.29745 |
|  |  |  |  |  |  |  |
| _cons | 20.33177 | 24.16059 | 2.53 | 0.011 | 1.980055 | 208.7723 |
|  |  |  |  |  |  |  |
| **Lower part** | **(base outcome)** |  |  |  |  |  |
|  |  |  |  |  |  |  |
| **Upper part** | |  |  |  |  |  |
| **sex** |  |  |  |  |  |  |
| **Male** | 0.837217 | 0.4838305 | -0.31 | 0.759 | 0.2697279 | 2.598665 |
|  |  |  |  |  |  |  |
| **Age category** |  |  |  |  |  |  |
| **<18** | 1 |  |  |  |  |  |
| **18-40** | 1.357171 | 1.147765 | 0.36 | 0.718 | 0.2586839 | 7.12032 |
| **41-60** | 0.6698523 | 0.6465279 | -0.42 | 0.678 | 0.1010221 | 4.441622 |
| **>60** | 0.1654972 | 0.2086945 | -1.43 | 0.154 | 0.013977 | 1.959606 |
|  |  |  |  |  |  |  |
| _cons | 0.1445242 | 0.1147915 | -2.44 | 0.015 | 0.0304687 | 0.6855297 |
|  |  |  |  |  |  |  |
| **Nodule** | (base outcome) |  |  |  |  |  |
|  |  |  |  |  |  |  |
| **Nodule-Oedema-Ulcer** | |  |  |  |  |  |
| **sex** |  |  |  |  |  |  |
| **Female** | 1 |  |  |  |  |  |
| **Male** | 0.8086913 | 0.7176187 | -0.24 | 0.811 | 0.14205 | 4.603884 |
|  |  |  |  |  |  |  |
| **Age group (in years)** | |  |  |  |  |  |
| **<18** | 1 |  |  |  |  |  |
| **18-40** | 1.557657 | 2.054159 | 0.34 | 0.737 | 0.1174763 | 20.65349 |
| **41-60** | 3.753085 | 5.856728 | 0.85 | 0.397 | 0.1762299 | 79.92769 |
| **>60** | 2.986585 | 4.670694 | 0.7 | 0.484 | 0.139312 | 64.02672 |
|  |  |  |  |  |  |  |
| _cons | 3.304955 | 4.062112 | 0.97 | 0.331 | 0.2971363 | 36.75998 |
|  |  |  |  |  |  |  |
| **Nodule-Ulcer** | |  |  |  |  |  |
| **sex** |  |  |  |  |  |  |
| **Female** | 1 |  |  |  |  |  |
| **Male** | 0.4065823 | 0.4117033 | -0.89 | 0.374 | 0.0558761 | 2.958497 |
|  |  |  |  |  |  |  |
| **Age group (in years)** | |  |  |  |  |  |
| **<18** | 1 |  |  |  |  |  |
| **18-40** | 1.005309 | 1.42692 | 0 | 0.997 | 0.0622468 | 16.2361 |
| **41-60** | 1.099804 | 1.913995 | 0.05 | 0.956 | 0.0363049 | 33.317 |
| **>60** | 1.965063 | 3.267559 | 0.41 | 0.685 | 0.075505 | 51.14193 |
|  |  |  |  |  |  |  |
| _cons | 2.801675 | 3.643397 | 0.79 | 0.428 | 0.2190208 | 35.83853 |
|  |  |  |  |  |  |  |
| **Oedema-Ulcer** | |  |  |  |  |  |
| **sex** |  |  |  |  |  |  |
| **Female** | 1 |  |  |  |  |  |
| **Male** | 1.28072 | 1.104771 | 0.29 | 0.774 | 0.236153 | 6.945684 |
|  |  |  |  |  |  |  |
| **Age category** |  |  |  |  |  |  |
| **<18** | 1 |  |  |  |  |  |
| **18-40** | 0.7904112 | 0.9720174 | -0.19 | 0.848 | 0.0709705 | 8.802949 |
| **41-60** | 1.825678 | 2.708978 | 0.41 | 0.685 | 0.0996309 | 33.45448 |
| **>60** | 2.513338 | 3.70816 | 0.62 | 0.532 | 0.1394422 | 45.30096 |
|  |  |  |  |  |  |  |
| _cons | 7.046525 | 8.04548 | 1.71 | 0.087 | 0.7518001 | 66.04617 |
|  |  |  |  |  |  |  |
| **Plaque** |  |  |  |  |  |  |
| **sex** |  |  |  |  |  |  |
| **Female** | 1 |  |  |  |  |  |
| **Male** | 6.57E-07 | 0.0006733 | -0.01 | 0.989 | 0 | . |
|  |  |  |  |  |  |  |
| **Age group (in years)** | |  |  |  |  |  |
| **<18** | 1 |  |  |  |  |  |
| **18-40** | 1.07864 | 5028.138 | 0 | 1 | 0 | . |
| **41-60** | 2.10E+07 | 8.54E+10 | 0 | 0.997 | 0 | . |
| **>60** | 2.711973 | 12838.95 | 0 | 1 | 0 | . |
|  |  |  |  |  |  |  |
| _cons | 1.17E-07 | 0.0004747 | 0 | 0.997 | 0 | . |
|  |  |  |  |  |  |  |
| **Plaque-Ulcer** | |  |  |  |  |  |
| **sex** |  |  |  |  |  |  |
| **Male** | 2.274967 | 3.395725 | 0.55 | 0.582 | 0.1220201 | 42.41497 |
|  |  |  |  |  |  |  |
| **Age group (in years)** | |  |  |  |  |  |
| **<18** | 1 |  |  |  |  |  |
| **18-40** | 1.24E-07 | 0.0002028 | -0.01 | 0.992 | 0 | . |
| **41-60** | 2.01E-07 | 0.0004301 | -0.01 | 0.994 | 0 | . |
| **>60** | 2.034329 | 3.81377 | 0.38 | 0.705 | 0.0516022 | 80.19993 |
|  |  |  |  |  |  |  |
| _cons | 0.6192924 | 1.048901 | -0.28 | 0.777 | 0.0223979 | 17.12314 |
|  |  |  |  |  |  |  |
| **Ulcer** |  |  |  |  |  |  |
| **sex** |  |  |  |  |  |  |
| **Male** | .515717 .470964 |  | -0.73 | 0.468 | 0.0861144 | 3.088498 |
|  |  |  |  |  |  |  |
| **Age group (in years)** | |  |  |  |  |  |
| **<18** | 1 |  |  |  |  |  |
| **18-40** | 1.67345 | 2.331957 | 0.37 | 0.712 | 0.1090088 | 25.69001 |
| **41-60** | 3.759608 | 6.135819 | 0.81 | 0.417 | 0.153444 | 92.11598 |
| **>60** | 4.933497 | 7.968301 | 0.99 | 0.323 | 0.208134 | 116.941 |
|  |  |  |  |  |  |  |
| _cons | 2.609283 | 3.378402 | 0.74 | 0.459 | 0.2062609 | 33.00847 |
|  |  |  |  |  |  |  |
| **Boil/Nodule** | (base outcome) |  |  |  |  |  |
|  |  |  |  |  |  |  |
| **Bruise/Injury** | |  |  |  |  |  |
| **sex** |  |  |  |  |  |  |
| **Female** | 1 |  |  |  |  |  |
| **Male** | 0.7626551 | 0.4036496 | -0.51 | 0.609 | 0.2702799 | 2.152002 |
|  |  |  |  |  |  |  |
| **Age group (in years)** | |  |  |  |  |  |
| **<18** | 1 |  |  |  |  |  |
| **18-40** | 0.5282765 | 0.4292437 | -0.79 | 0.432 | 0.1074557 | 2.597128 |
| **41-60** | 1.047511 | 0.8806716 | 0.06 | 0.956 | 0.2016181 | 5.442363 |
| **>60** | 0.5950995 | 0.5131334 | -0.6 | 0.547 | 0.1098066 | 3.225156 |
|  |  |  |  |  |  |  |
| _cons | 1.414619 | 1.010068 | 0.49 | 0.627 | 0.3490302 | 5.733446 |
|  |  |  |  |  |  |  |
| **Don’t Know** | |  |  |  |  |  |
| **sex** |  |  |  |  |  |  |
| **Female** | 1 |  |  |  |  |  |
| **Male** | 1.015172 | 0.4671315 | 0.03 | 0.974 | 0.4119643 | 2.501613 |
|  |  |  |  |  |  |  |
| **Age group (in years)** | |  |  |  |  |  |
| **<18** | 1 |  |  |  |  |  |
| **18-40** | 1.467433 | 1.116653 | 0.5 | 0.614 | 0.330241 | 6.520574 |
| **41-60** | 0.9133223 | 0.7743355 | -0.11 | 0.915 | 0.1733628 | 4.811629 |
| **>60** | 1.500702 | 1.202305 | 0.51 | 0.612 | 0.3121362 | 7.21514 |
|  |  |  |  |  |  |  |
| _cons | 1.240769 | 0.8784658 | 0.3 | 0.761 | 0.3097702 | 4.969841 |
|  |  |  |  |  |  |  |
| **Itch/Rush** |  |  |  |  |  |  |
| **sex** |  |  |  |  |  |  |
| **Female** | 1 |  |  |  |  |  |
| **Male** | 0.5349048 | 0.3341959 | -1 | 0.317 | 0.1572049 | 1.820066 |
|  |  |  |  |  |  |  |
| **Age group (in years)** | |  |  |  |  |  |
| **<18** | 1 |  |  |  |  |  |
| **18-40** | 1.305205 | 1.646301 | 0.21 | 0.833 | 0.1101609 | 15.46428 |
| **41-60** | 2.382302 | 3.062978 | 0.68 | 0.5 | 0.191685 | 29.60775 |
| **>60** | 4.418123 | 5.399723 | 1.22 | 0.224 | 0.4026429 | 48.47922 |
|  |  |  |  |  |  |  |
| _cons | 0.3238649 | 0.3713059 | -0.98 | 0.325 | 0.0342354 | 3.063746 |
|  |  |  |  |  |  |  |
| **Swells/Oedema** | |  |  |  |  |  |
| **sex** |  |  |  |  |  |  |
| **Female** | 1 |  |  |  |  |  |
| **Male** | 1.294871 | 0.7071223 | 0.47 | 0.636 | 0.4440072 | 3.776271 |
|  |  |  |  |  |  |  |
| **Age group (in years)** | |  |  |  |  |  |
| **<18** | 1 |  |  |  |  |  |
| **18-40** | 1.009316 | 1.010182 | 0.01 | 0.993 | 0.1419368 | 7.177266 |
| **41-60** | 2.527626 | 2.535813 | 0.92 | 0.355 | 0.3537969 | 18.05808 |
| **>60** | 2.016043 | 2.017807 | 0.7 | 0.484 | 0.2835001 | 14.3366 |
|  |  |  |  |  |  |  |
| _cons | 0.4368065 | 0.399072 | -0.91 | 0.365 | 0.0728821 | 2.617928 |

*Results of multinomial Logistics regression table for Buruli ulcer lesion presentation from Ghana by sex and age groups.

* All data with 0 or low values (< 10) in the analysis were ignored. P value of <025 (a more relaxed cut-off) was selected to avoid prematurely excluding predictors that are not statistically significant as no predictors met the p < 0.05 threshold in the provided regression outputs.

Primary outcome: The type of lesion (Nodule, oedema, plaque and ulcer), Location of lesion (Lower and Upper part), disease progression (Nodule-oedema-ulcer, nodule- ulcer, oedema-ulcer, plaque, plaque-ulcer, ulcer), and initiation of lesion (boil/nodule, don’t know, itch/rash, swell /oedema).

Independent variables: Age groups and sex
