# Supplementary material for: Comparative analysis of Buruli ulcer in Ghana and Côte d’Ivoire: A cross-sectional study
Source: PLoS Negl Trop Dis. 2026 Jan 12;20(1):e0013912. doi: 10.1371/journal.pntd.0013912 (PMC12822952; doi:10.1371/journal.pntd.0013912)
Supplement: S5 Table — (DOCX) [file pntd.0013912.s005.docx]

**S5 Table: Comparison of BU Lesion Characteristics, Between Côte d’Ivoire and Ghana (Chi-Square Analysis)**

|  | **Côte d’Ivoire.** | **Côte d’Ivoire.** | **Ghana** | **Ghana** | **Both Countries** | **Total** |
| --- | --- | --- | --- | --- | --- | --- |
|  | No. | % | No. | % | No. | % |
| **Lesion Presentation** |  |  |  |  |  |  |
| Nodule | 0 | 0 | 4 | 3.8 | 4 | 1.3 |
| Oedema | 44 | 22.4 | 22 | 21.2 | 66 | 22 |
| Plaque | 23 | 11.7 | 2 | 1.9 | 25 | 8.3 |
| Ulcer | 129 | 65.8 | 76 | 73.1 | 205 | 68.3 |
| Total | 196 | 100 | 104 | 100 | 300 | 100 |
|  |  |  |  |  |  |  |
| Pearson chi2(3) = 15.9637 Pr = 0.001 |  |  |  |  |  |  |
|  |  |  |  |  |  |  |
| **Category of lesion** |  |  |  |  |  |  |
| I | 19 | 9.7 | 27 | 26 | 46 | 15.3 |
| II | 133 | 67.9 | 35 | 33.7 | 168 | 56 |
| III | 44 | 22.4 | 42 | 40.4 | 86 | 28.7 |
| Total | 196 | 100 | 104 | 100 | 300 | 100 |
|  |  |  |  |  |  |  |
| Pearson chi2(2) = 33.5460 Pr = 0.000 |  |  |  |  |  |  |
|  |  |  |  |  |  |  |
| **Location of lesion** |  |  |  |  |  |  |
| Arm | 36 | 18.4 | 7 | 6.7 | 43 | 14.3 |
| Cheek | 2 | 1 | 1 | 1 | 3 | 1 |
| Foot | 105 | 53.6 | 21 | 20.2 | 126 | 42 |
| Genitalia | 3 | 1.5 | 0 | 0 | 3 | 1 |
| Leg | 30 | 15.3 | 72 | 69.2 | 102 | 34 |
| Thigh | 10 | 5.1 | 1 | 1 | 11 | 3.7 |
| Trunk | 8 | 4.1 | 2 | 1.9 | 10 | 3.3 |
| Whole body | 2 | 1 | 0 | 0 | 2 | 0.7 |
| Total | 196 | 100 | 104 | 100 | 300 | 100 |
|  |  |  |  |  |  |  |
| Pearson chi2(7) = 89.3376 Pr = 0.000 |  |  |  |  |  |  |
|  |  |  |  |  |  |  |
| **Location of lesion (Upper or lower)** |  |  |  |  |  |  |
| Both | 2 | 1 | 0 | 0 | 2 | 0.7 |
| Lower part | 148 | 75.5 | 94 | 90.4 | 242 | 80.7 |
| Upper part | 46 | 23.5 | 10 | 9.6 | 56 | 18.7 |
| Total | 196 | 100 | 104 | 100 | 300 | 100 |
|  |  |  |  |  |  |  |
| Pearson chi2(2) = 9.9112 Pr = 0.007 |  |  |  |  |  |  |
|  |  |  |  |  |  |  |
| **Stages of Disease Progression** |  |  |  |  |  |  |
| Nodule | 0 | 0 | 5 | 4.8 | 5 | 1.7 |
| Nodule-Oedema-Ulcer | 0 | 0 | 29 | 27.9 | 29 | 9.7 |
| Nodule-Ulcer | 17 | 8.7 | 8 | 7.7 | 25 | 8.3 |
| Oedema | 5 | 2.6 | 0 | 0 | 5 | 1.7 |
| Oedema-Ulcer | 42 | 21.4 | 41 | 39.4 | 83 | 27.7 |
| Plaque | 1 | 0.5 | 0 | 0 | 1 | 0.3 |
| Plaque-Ulcer | 22 | 11.2 | 3 | 2.9 | 25 | 8.3 |
| Ulcer | 109 | 55.6 | 18 | 17.3 | 127 | 42.3 |
| Total | 196 | 100 | 104 | 100 | 300 | 100 |
|  |  |  |  |  |  |  |
| Pearson chi2(7) = 104.5122 Pr = 0.000 |  |  |  |  |  |  |
|  |  |  |  |  |  |  |
| **Method of Contraction** |  |  |  |  |  |  |
| Boil/Nodule | 3 | 1.5 | 22 | 21.2 | 25 | 8.3 |
| Bruise/Injury | 31 | 15.8 | 23 | 22.1 | 54 | 18 |
| Dont't Know | 145 | 74 | 24 | 23.1 | 169 | 56.3 |
| Itch/Rush | 1 | 0.5 | 15 | 14.4 | 16 | 5.3 |
| Swells/Oedema | 16 | 8.2 | 20 | 19.2 | 36 | 12 |
| Total | 196 | 100 | 104 | 100 | 300 | 100 |
|  |  |  |  |  |  |  |
| Pearson chi2(4) = 95.7436 Pr = 0.000 |  |  |  |  |  |  |
