# Supplementary material for: Comparative analysis of Buruli ulcer in Ghana and Côte d’Ivoire: A cross-sectional study
Source: PLoS Negl Trop Dis. 2026 Jan 12;20(1):e0013912. doi: 10.1371/journal.pntd.0013912 (PMC12822952; doi:10.1371/journal.pntd.0013912)
Supplement: S6 Table — (DOCX) [file pntd.0013912.s008.docx]

S6 Table: Treatment Seeking Behaviour Among the BU in Ghana and Côte d’Ivoire by Age Group

|  |  |  | **Côte d’Ivoire** | |  |  |  |  |  |  | **Ghana** | |  |  |  |  |
| --- | --- | --- | --- | --- | --- | --- | --- | --- | --- | --- | --- | --- | --- | --- | --- | --- |
| **Age Groups** | **<15 yrs** |  | **(15-60)yrs** |  | **>60yrs** |  | **Total** |  |  | **<15yrs** |  | **(15-60)yrs** |  | **>60yrs** |  | **Total** |
|  | **n(%)** |  | **n(%)** |  | **n(%)** |  |  |  |  | **n(%)** |  | **n(%)** |  | **n(%)** |  |  |
| **Treatment options** |  |  |  |  |  |  |  |  |  |  |  |  |  |  |  |  |
| Clinic/hospital | 33(19.1) |  | 131(75.7) |  | 9(5.2) |  | 173(100) |  |  | 5(12.2) |  | 24(58.5) |  | 12(29.3) |  | 41(100) |
| Clinic+Traditional | 4(33.3) |  | 8(66.7) |  | 0(0.0) |  | 12(100) |  |  | 0(0.0) |  | 1(100.0) |  | 0(0.0) |  | 1(100) |
| Traditional | 0(0.0) |  | 0)0.0) |  | 0(0.0) |  | 0(0.0) |  |  | 2(6.9) |  | 16(55.2) |  | 11(37.9) |  | 29(100) |
| Traditional+Self treatment | 0(0.0) |  | 0(0.0) |  | 0(0.0) |  | 0(0.0) |  |  | 1(25) |  | 0(0.0) |  | 3(75.0) |  | 4(100) |
| Clinic+Self-treatment | 1(7.7) |  | 12(92.3) |  | 0(0.0) |  | 13(100) |  |  | 2(6.7) |  | 20(66.7) |  | 8(26.7) |  | 30(100) |
| Self-treatment | 0(0.0) |  | 0(0.0) |  | 0(0.0) |  | 0(0.0) |  |  | 0(0.0) |  | 0(0.0) |  | 0(0.0) |  | 0(0.0) |
| Total | 38(19.2) |  | 151(76.3) |  | 9(4.5) |  | 198(100) |  |  | 10(9.5) |  | 61(58.1) |  | 34(32.4) |  | 105(100) |
| **Antibiotic treatment** |  |  |  |  |  |  |  |  |  |  |  |  |  |  |  |  |
| No | 0(0.0) |  | 0(0.0) |  | 0(0.0) |  | 0(0.0) |  |  | 5(8.9) |  | 32(57.1) |  | 19(33.9) |  | 56 |
| Yes | 38(19.2) |  | 151(76.3) |  | 9(4.5) |  | 198(100.0) |  |  | 5(10.2) |  | 29(59.2) |  | 15(30.6) |  | 49 |
| Total | 38(19.2) |  | 151(76.3) |  | 9(4.5) |  | 198(100.0) |  |  | 10(9.5) |  | 61(58.1) |  | 34(32.4) |  | 105 |
| **Antibiotic type** |  |  |  |  |  |  |  |  |  |  |  |  |  |  |  |  |
| None | 0(0.0) |  | 0(0.0) |  | 0(0.0) |  | 0(0.0) |  |  | 5(8.9) |  | 32(57.1) |  | 19(33.9) |  | 56 |
| Clarithromycin+Rifampicin | 0(0.0) |  | 0(0.0) |  | 0(0.0) |  | 0(0.0) |  |  | 0(0.0) |  | 0(0) |  | 1(100.0) |  | 1 |
| Streptomycin+Rifampicin | 38(19.2) |  | 151(76.3) |  | 9(4.5) |  | 198(100.0) |  |  | 2(50.0) |  | 2(50) |  | 0(0.0) |  | 4 |
| Other Antibiotics | 0(0) |  | 0(0) |  | 0(0) |  | 0(0) |  |  | 3(6.8) |  | 27(61.4) |  | 14(31.8) |  | 44 |
| Total | 38(19.2) |  | 151(76.3) |  | 9(4.5) |  | 198(100.0) |  |  | 10(9.5) |  | 61(58.1) |  | 34(32.4) |  | 105 |
| **Duration of treatment** |  |  |  |  |  |  |  |  |  |  |  |  |  |  |  |  |
| <1 week | 1(6.3) |  | 14(87.5) |  | 1(6.3) |  | 16 |  |  | 0(0) |  | 3(75.0) |  | 1(25.0) |  | 4 |
| 1-2 weeks | 7(24.1) |  | 20(69) |  | 2(6.9) |  | 29 |  |  | 4(30.8) |  | 4(30.8) |  | 5(38.5) |  | 13 |
| 3-4 weeks | 8(19.5) |  | 29(70.7) |  | 4(9.8) |  | 41 |  |  | 0(0.0) |  | 9(75.0) |  | 3(25.0) |  | 12 |
| 5-6 weeks | 0(0.0) |  | 0(0.0) |  | 0(0.0) |  | 0 |  |  | 0(0.0) |  | 4(100.0) |  | 0(0.0) |  | 4 |
| 7-8 weeks | 2(11.8) |  | 15(88.2) |  | 0(0.0) |  | 17 |  |  | 1(16.7) |  | 3(50.0) |  | 2(33.3) |  | 6 |
| above 8 Weeks | 17(23.9) |  | 53(74.6) |  | 1(1.4) |  | 71 |  |  | 5(9.8) |  | 31(60.8) |  | 15(29.4) |  | 51 |
| Don't know | 3(12.5) |  | 20(83.3) |  | 1(4.2) |  | 24 |  |  | 0(0.0) |  | 7(46.7) |  | 8(53.3) |  | 15 |
| Total | 38(19.2) |  | 151(76.3) |  | 9(4.5) |  | 198 |  |  | 10(9.5) |  | 61(58.1) |  | 34(32.4) |  | 105 |
