# Supplementary figures and images for: Comparative analysis of Buruli ulcer in Ghana and Côte d’Ivoire: A cross-sectional study
Source: PLoS Negl Trop Dis. 2026 Jan 12;20(1):e0013912. doi: 10.1371/journal.pntd.0013912 (PMC12822952; doi:10.1371/journal.pntd.0013912)

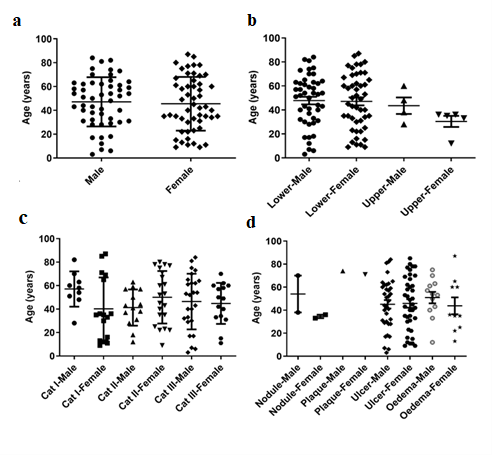

Supplement: S1 Fig — (TIFF) [file pntd.0013912.s006.tiff]

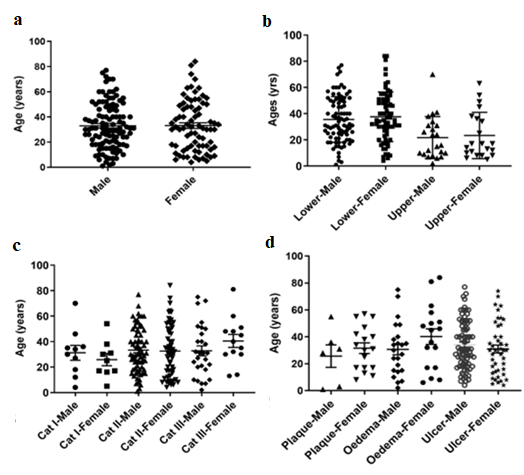

Supplement: S2 Fig — (TIF) [file pntd.0013912.s007.tif]
